# CDK5RAP3 Regulates Testosterone Production in Mouse Leydig Cells

**DOI:** 10.3390/ijms27020586

**Published:** 2026-01-06

**Authors:** Jian Ruan, Qianyi Dong, Yufan Jin, Yuhong Yang, Jun Li, Yafei Cai

**Affiliations:** 1 College of Life Sciences, Anhui Normal University, Wuhu 241000, China; 2 College of Animal Science and Technology, Nanjing Agricultural University, Nanjing 210095, China

**Keywords:** testosterone, Leydig cells, CDK5RAP3, CEBPB, SMAD4

## Abstract

Testosterone (T) produced by Leydig cells (LCs) is essential for male reproduction; yet, the regulatory mechanisms underlying steroidogenesis remain incompletely understood. Here, we investigated the role of cyclin-dependent kinase 5 regulatory subunit-associated protein 3 (CDK5RAP3) in Leydig cell development and steroidogenesis, based on its identification by immunoprecipitation-mass spectrometry (IP-MS) as a protein associated with steroidogenesis and cholesterol metabolism in mouse testicular tissue. Using human samples, we found that CDK5RAP3 expression was significantly reduced in Leydig cells from patients with spermatogenic failure (T < 10.4 nmol/L). Notably, CDK5RAP3 expression increased during mouse postnatal Leydig cell maturation and regeneration in an ethane dimethanesulfonate (EDS)-induced rat model. Functional analyses in primary LCs and MLTC-1 cells showed that hCG stimulation triggered CDK5RAP3 nuclear translocation without altering its overall expression, while CDK5RAP3 knockdown markedly impaired hCG-induced testosterone production and reduced the expression of the steroidogenic regulator steroidogenic acute regulatory (STAR) protein, as well as key steroidgenic enzymes, including cytochrome P450 family 11 subfamily A member 1 (CYP11A1), 17a-hydroxylase (CYP17A1), and 3β-hydroxysteroid dehydrogenase (HSD3B). Conversely, CDK5RAP3 overexpression enhanced testosterone production in the absence of hCG. In vivo, AAV2/9-mediated CDK5RAP3 silencing in adult mouse testes resulted in a significant reduction in serum testosterone levels compared with controls (3.60 ± 0.38 ng/mL vs. 1.83 ± 0.37 ng/mL). Mechanistically, CDK5RAP3 interacted with SMAD4 and CEBPB, and BMP pathway inhibition by Noggin rescued the testosterone deficit caused by CDK5RAP3 loss. Together, these findings identify CDK5RAP3 as an essential regulator of Leydig cell steroidogenesis and provide insight into its potential relevance to male infertility associated with low testosterone.

## 1. Introduction

Testosterone is essential for maintaining male secondary sexual characteristics and supporting normal spermatogenesis. Testosterone deficiency is frequently associated with late-onset hypogonadism, sexual dysfunction, and impaired fertility [1,2,3]. In males, testosterone is primarily synthesized and secreted by testicular LCs, a process regulated by the steroidogenic regulator STAR and steroidogenic enzymes, including CYP11A1, CYP17A1, and HSD3B [4]. Luteinizing hormone (LH) or human chorionic gonadotropin (hCG) activate the cyclic adenosine monophosphate (cAMP) signaling pathway, triggering a cascade that promotes testosterone synthesis in LCs [5].

During mammalian development, LCs progress through four distinct stages: Stem Leydig cells (SLCs), Progenitor Leydig cells (PLCs), Immature Leydig cells (ILCs), and Adult Leydig cells (ALCs). Undifferentiated SLCs have unlimited self-renewal potential and differentiate into PLCs, which begin to produce low levels of androgens. PLCs further differentiate into ILCs, which exhibit an enhanced testosterone synthetic capacity. ILCs eventually mature into ALCs, which are highly efficient in testosterone production [6]. This differentiation process is accompanied by coordinated changes in the steroidogenic gene and transcriptional factors [7]. As a bZIP-type transcription factor, CCAAT/enhancer-binding protein beta (CEBPB) plays a critical role in LH-regulated Leydig cell differentiation and function, interacting with transcription factors such as cAMP response element-binding protein–binding protein (CBP) and NR5A1 to directly regulate steroid hormone synthesis [8,9,10]. CBP is a transcriptional co-activator with intrinsic histone acetyltransferase activity and has been reported to interact with CDK5RAP3 [11,12]. SMAD4, a central mediator of TGF-β signaling, has been implicated in the regulation of testicular development and Leydig cell function [13,14].

CDK5RAP3, also known as C53 or LZAP, is an ARF-binding protein containing Leucine-X-Leucine-Leucine-Leucine (LXXLL) motifs and a leucine zipper domain, and is broadly expressed across multiple tissues and organs. Structurally, the canonical CDK5RAP3 transcript is 1841 bp in length and encodes a 506-amino acid protein with a molecular weight of approximately 57 kDa [15]. Although CDK5RAP3 contains a leucine zipper domain for protein dimerization and two LXXLL motifs involved in transcription factor interactions, it lacks other well-characterized functional domains or enzymatic active sites [16]. LXXLL motifs are crucial protein sequences in coactivators like SRC-1, CBP, and TReP-132, mediating their binding to nuclear receptors (like SF-1, ER, and AR) to regulate steroid hormone gene expression [17,18]. CDK5RAP3 participates in diverse cellular processes, including autophagy, endoplasmic reticulum stress, and cell cycle regulation, thereby contributing to both development and tumorigenesis [19,20,21,22,23]. However, its functional role in testicular cells remains poorly understood.

In this study, we aim to investigate the potential role of CDK5RAP3 in Leydig cell development and steroidogenesis. Specifically, we sought to examine whether CDK5RAP3 is associated with stage-dependent Leydig cell maturation, responsive to LH/hCG signaling, and involved in the transcriptional regulation of genes related to Leydig cell development and steroidogenesis.

## 2. Results

### 2.1. CDK5RAP3 Is Closely Associated with Steroidogenesis in Testis

**Figure 1:**
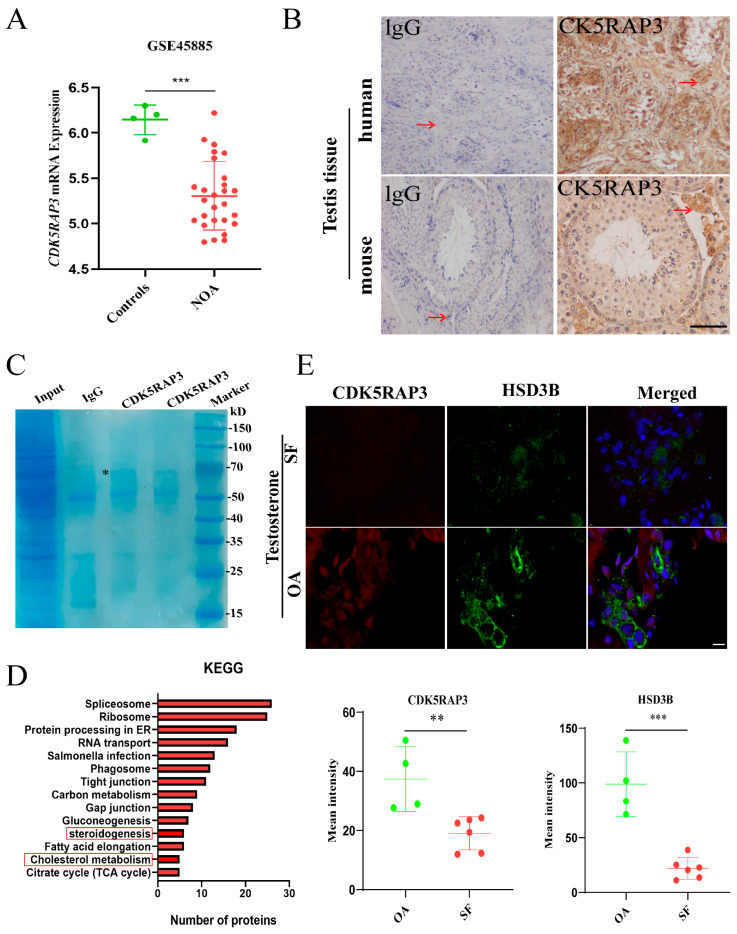
The strong correlation of CDK5RAP3 with testicular Leydig cell steroidogenesis. (**A**) Expression levels of *CDK5RAP3* mRNA in testicular tissues of NOA patients (*n* = 27) and controls (*n* = 4) in the GSE45885 dataset. (**B**) IHC result in human and mouse testicular tissue. The arrows indicate Leydig cells located in the interstitial space of the testes. Scale bar, 50 µm. (**C**) SDS-PAGE analysis of eluted proteins bound to CDK5RAP3 antibodies from Protein A/G magnetic beads, followed by Coomassie brilliant blue staining; * is CDK5RAP3. (**D**) KEGG pathway enrichment analysis of proteomics data identifying proteins interacting with CDK5RAP3. The red box highlights the steroidgenesis and cholesterol metabolism pathway, which is directly related to steroidogenesis and provides essential substrates for testosterone biosynthesis. (**E**) Immunofluorescence results of CDK5RAP3 and HSD3B in leydig cells of OA (*n* = 4) and SF (*n* = 6) patients. Scale bar, 10 µm. ** *p* < 0.01, *** *p* < 0.001. Abbreviations: CDK5RAP3, cyclin-dependent kinase 5 regulatory subunit–associated protein 3; NOA, non-obstructive azoospermia; OA, obstructive azoospermia; IHC, immunohistochemistry; SDS-PAGE, sodium dodecyl sulfate–polyacrylamide gel electrophoresis; HSD3B, 3β-hydroxysteroid dehydrogenase; SF, spermatogenic failure.

An analysis of transcriptomic data from the GEO database (GSE45885), comprising samples from 4 normozoospermic (Controls, normal spermatogenesis) and 27 non-obstructive azoospermic (NOA) individuals, revealed a significant downregulation of *CDK5RAP3* transcript levels in NOA compared to normozoospermia (*p* < 0.001) (Figure 1A). This finding indicates that the reduced *CDK5RAP3* expression may be linked to testicular dysfunction. To examine the cellular localization of CDK5RAP3 in the testicular microenvironment, an immunohistochemistry (IHC) analysis was performed on human and mouse testicular tissues. As shown in Figure 1B, CDK5RAP3 was broadly expressed in various cell types within human testicular sections, whereas its expression in mouse testis was predominantly localized to LCs.

The subsequent interactome profiling via IP-MS of mouse testicular lysates identified CDK5RAP3-interacting protein networks (Figure 1C), and a KEGG pathway analysis revealed significant enrichment in pathways related to steroidogenesis and cholesterol homeostasis mechanisms (*p* < 0.05) (Figure 1D).

To test this hypothesis, we performed immunofluorescence analysis on clinical testicular specimens from obstructive azoospermia (OA) patients (control, T > 10.4 nmol/L) and patients with spermatogenic failure (SF) (T < 10.4 nmol/L). The fluorescence intensity of CDK5RAP3 in LCs was significantly reduced in spermatogenic failure patients (*p* < 0.05) (Figure 1E), and showed a strong positive correlation with HSD3B expression.

### 2.2. Expression Pattern of CDK5RAP3 in Mouse Testis Leydig Cells at Different Postnatal Ages

**Figure 2:**
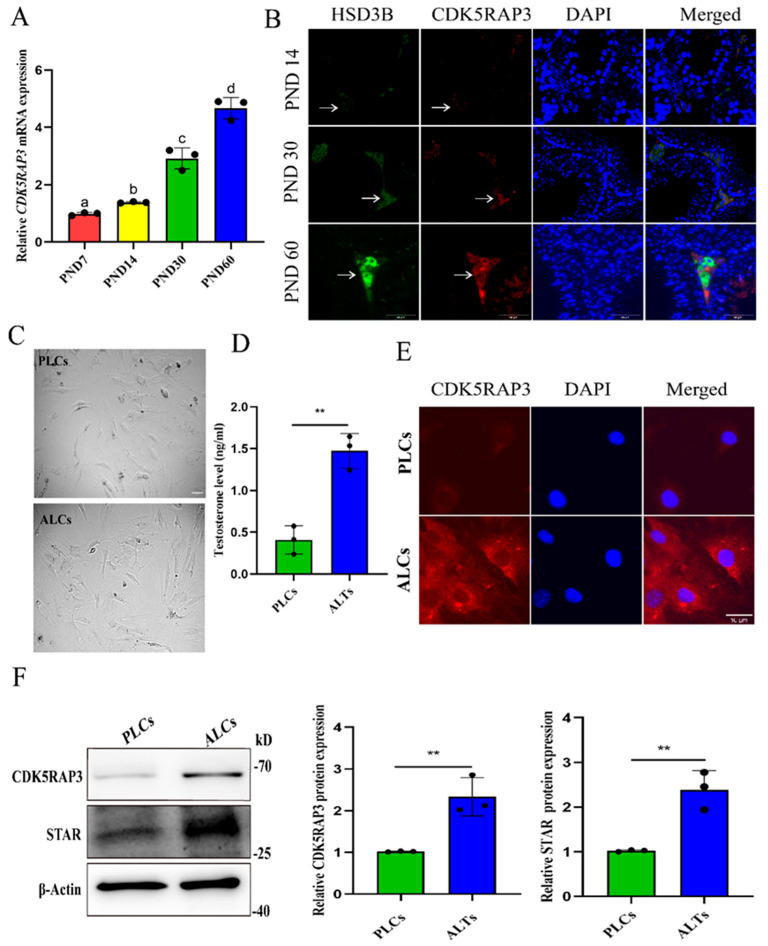
The expression pattern of CDK5RAP3 in the testes of mice at different ages. (**A**) *CDK5RAP3* mRNA levels in mouse testes at different postnatal ages. Different letters indicate significant differences. *p* < 0.05. (*n* = 3 per group). (**B**) Immunofluorescence results of CDK5RAP3 and HSD3B in testicular tissue sections of different age. The arrows indicate Leydig cells located in the interstitial space of the testes. Scale bar: 50 µm. (**C**) Bright-field images of primary testicular PLCs and ALCs. Scale bar, 100 µm. (**D**) Testosterone levels in PLCs and ALCs. (**E**) Immunofluorescence results of CDK5RAP3 in PLCs and ALCs. Scale bar: 10 µm. (**F**) Western blotting results of CDK5RAP3 and STAR in PLCs and ALCs. ** *p* < 0.01. Data represent at least three independent biological replicates. Abbreviations: PLCs, progenitor Leydig cells; ALCs, adult Leydig cells; STAR, steroidogenic acute regulatory protein.

To characterize the developmental expression pattern of CDK5RAP3 during mouse testicular maturation, qRT-PCR revealed a gradual increase in *CDK5RAP3* mRNA levels from testes of postnatal day 7 (PND7) to testes of sexual maturity (PND60) (Figure 2A). Dual immunofluorescence staining demonstrated the synchronized spatiotemporal expression patterns of CDK5RAP3 and the steroidogenic marker HSD3B, with both showing Leydig cell-specific localization and a positive correlation with postnatal age (Figure 2B).

To dissect CDK5RAP3 regulation in Leydig cell development, we established purified primary PLCs from prepubertal (2–3 weeks) mice versus ALCs from mature (7–8 weeks) mice (Figure 2C). The ELISA results identified a significant increase in testosterone secretion in ALCs compared to PLCs (*p* < 0.05) (Figure 2D), which was accompanied by the marked cytoplasmic enrichment and elevated fluorescence intensity of CDK5RAP3 in ALCs (Figure 2E). A Western blot analysis further confirmed the significant upregulation of both CDK5RAP3 and STAR in ALCs compared to PLCs (*p* < 0.05) (Figure 2F).

### 2.3. Temporal Expression Dynamics of CDK5RAP3 During Leydig Cell Regeneration

**Figure 3:**
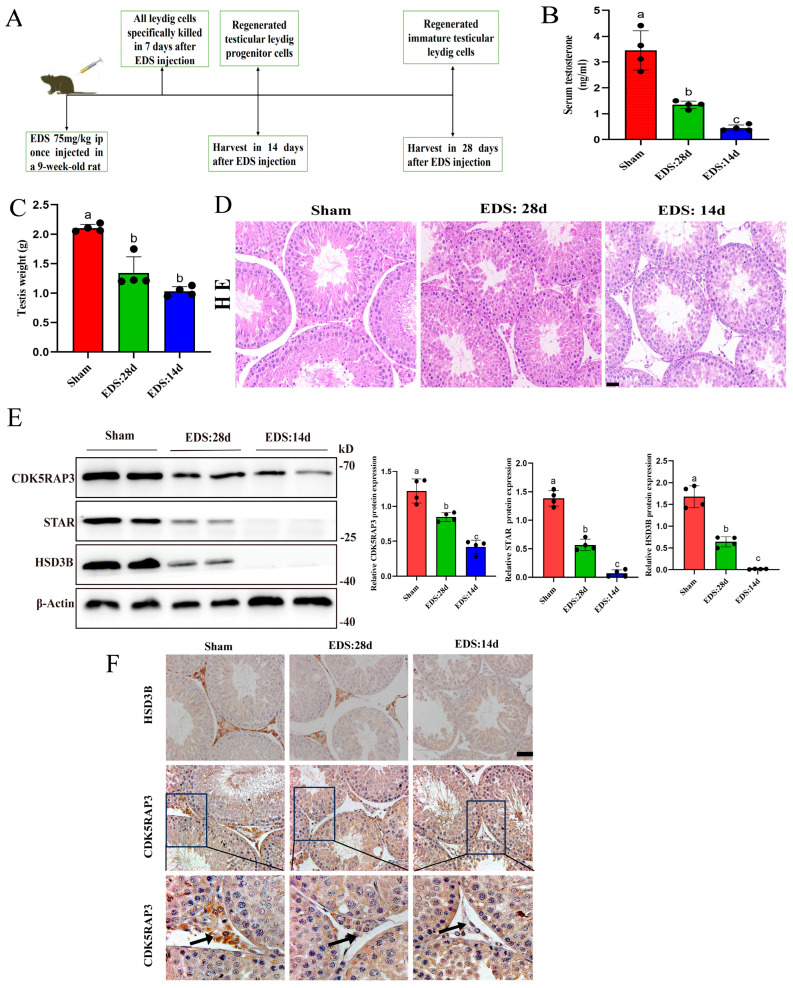
The results in the rat EDS-induced model (*n* = 4 per group). (**A**) Experimental procedure of the rat EDS-induced model. (**B**) Statistical analysis of serum testosterone in the rat EDS-induced model. (**C**) Statistical analysis of testis weight in the rat EDS-induced model. (**D**) HE staining of testis in the rat EDS-induced model. Scale bar, 20 µm. (**E**) Western blotting results of testicular tissue from the rat EDS-induced model. (**F**) IHC results of testicular tissue from the rat EDS-induced model. The arrows indicate CDK5RAP3-positive Leydig cells located in the interstitial space of the testes. Scale bar, 10 µm. Different letters indicate significant differences, *p* < 0.05. Abbreviations: EDS, ethane dimethanesulfonate.

To elucidate the in vivo regulatory mechanisms by which CDK5RAP3 governs Leydig cell lineage differentiation, reflecting the Leydig cell regeneration character and progressive steroidogenic capacity, we euthanized the rats at post-treatment day 14 (PTD14) and PTD28 after EDS Intraperitoneal injection (Figure 3A); this time point corresponds to the phase of Leydig cell regeneration following EDS treatment based on the established morphological and marker-based criteria [24]. Then, we quantitatively assessed the serum testosterone levels (Figure 3B), testicular weight (Figure 3C), and histological features using HE staining (Figure 3D). In EDS-treated rats, the serum testosterone dropped to near-undetectable levels by PTD14, accompanied by a significant decline in testicular weight (*p* < 0.05) and only a small amount of oval interstitial cells. By PTD28, both the Leydig cell numbers and serum testosterone levels partially recovered, although they remained significantly lower compared to age-matched controls (*p* < 0.05).

As demonstrated by TUNEL staining (Figure S1), seminiferous tubules exhibited increasing numbers of apoptotic germ cells in response to systemic testosterone deficiency (*p* < 0.05). The Western blot analysis revealed the synchronized temporal expression patterns of CDK5RAP3 and steroidogenic markers HSD3B and StAR. The CDK5RAP3 expression reached its lowest point at PTD14. Differentiation into regenerating immature Leydig cells (RILCs) by PTD28 led to a marked rebound in CDK5RAP3 expression, although levels remained below those observed in untreated controls (Figure 3E). The immunohistochemical analysis confirmed these temporal changes: CDK5RAP3 staining was significantly reduced in regenerating progenitor Leydig cells (RPLCs) but gradually increased during RILC maturation, paralleling the expression pattern of HSD3B. In untreated testes, CDK5RAP3 showed strong immunoreactivity, consistent with its physiological role in mature Leydig cell function (Figure 3F). Collectively, these findings identify CDK5RAP3 as a key regulator of Leydig cell lineage specification and steroidogenic capacity.

### 2.4. AAV2/9-shCDK5RAP3-Mediated Knockdown of CDK5RAP3 Expression in Mouse Testis

**Figure 4:**
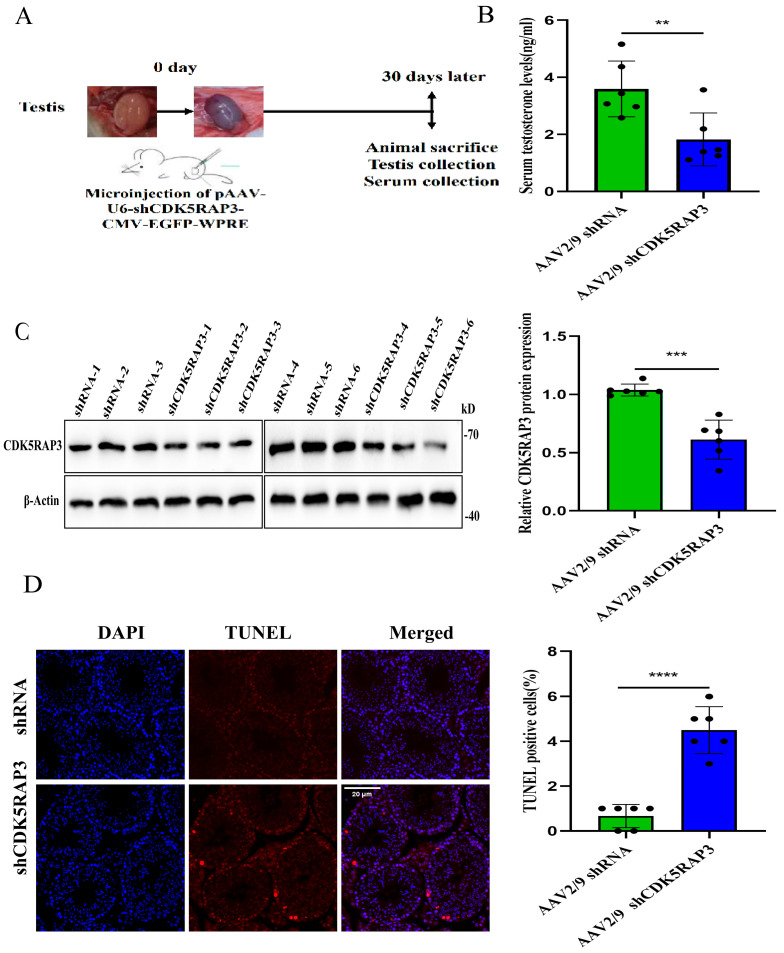
In vivo injection of AAV2/9-shCDK5RAP3 (*n* = 6 per group). (**A**) Schematic of the procedure for in situ testicular injection of AAV virus. (**B**) Serum testosterone levels in mice. (**C**) Western blot results of CDK5RAP3 in mouse testes. (**D**) TUNEL results in mouse testicular tissue. Scale bar, 20 µm. ** *p* < 0.01. *** *p* < 0.001. **** *p* < 0.0001. Abbreviations: TUNEL, terminal deoxynucleotidyl transferase-mediated dUTP nick end labeling.

To investigate the regulatory function of CDK5RAP3 on testosterone biosynthesis in vivo, the bilateral intratesticular administration of AAV2/9-shCDK5RAP3 was performed as outlined in Figure 4A [25]. At the 30-day post-injection endpoint, systemic blood sampling was performed via removing the eyeball followed by bilateral epididymal and testicular tissue harvesting. A testicular cross-sections assay demonstrated cell-type-specific EGFP expression patterns, with intense fluorescence signals localized to Leydig cell clusters, and the seminiferous tubule epithelium remained EGFP-negative (Figure S2A). A subsequent quantitative assessment of sperm counts through Neubauer hemocytometer analysis revealed comparable sperm counts between the shRNA control and CDK5RAP3 knockdown cohorts (Figure S2B). Consistent with these findings, the testicular weight showed no statistically significant intergroup variation (Figure S2C).

There was a significant reduction in serum testosterone concentrations in the AAV2/9-shCDK5RAP3 cohort relative to the shRNA controls (*p* < 0.05, Figure 4B). A Western blot quantification revealed successful CDK5RAP3 knock-down at the protein level, with a significant downregulation in the AAV2/9-shCDK5RAP3 group compared to the AAV2/9-shRNA control group (*p* < 0.05, Figure 4C). Immunofluorescence confirmed that the CDK5RAP3 signal intensity was markedly reduced in the LCs of the AAV2/9-shCDK5RAP3 group relative to the control group (*p* < 0.05, Figure S2D).

TUNEL (terminal deoxynucleotidyl transferase–mediated dUTP nick end labeling) analysis revealed a significant increase in apoptotic cells within the seminiferous tubules of the AAV2/9-shCDK5RAP3 group compared with controls (*p* < 0.05) (Figure 4D).

### 2.5. hCG Treatment Affects CDK5RAP3 Expression in Primary LCs and MLTC-1 Cells

**Figure 5:**
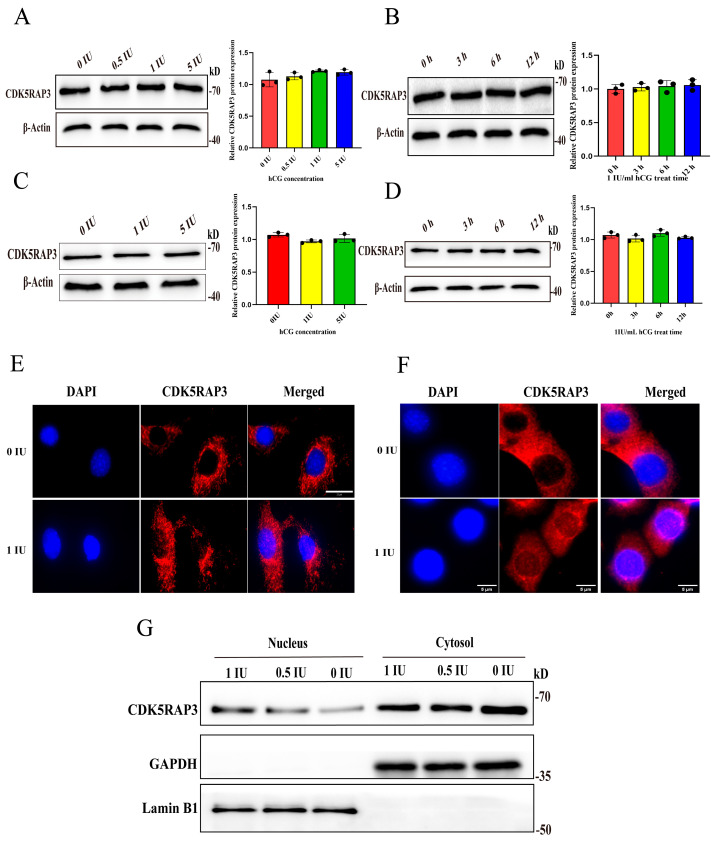
The effect of hCG on CDK5RAP3 expression in primary LCs and MLTC-1 cells. (**A**) The Western blot results of CDK5RAP3 in primary LCs after treatment with different concentrations of hCG. (**B**) The Western blotting results of CDK5RAP3 in primary LCs after treatment with 1 IU/mL hCG for different durations. (**C**) The Western blot results of CDK5RAP3 in MLTC-1 after treatment with different concentrations of hCG. (**D**) The Western blotting results of CDK5RAP3 in MLTC-1 after treatment with 1 IU/mL hCG for different durations. (**E**) Immunofluorescence results of nuclear translocation of CDK5RAP3 stimulated by hCG in primary LCs. Scale bar, 20 µm. (**F**) Immunofluorescence results of nuclear translocation of CDK5RAP3 stimulated by hCG in MLTC-1 cells. Scale bar, 5 µm. (**G**) Nuclear-cytoplasmic fractionation result of CDK5RAP3 in MLTC-1 cells induced by hCG stimulation. Data represent at least three independent biological replicates. Abbreviations: hCG, human chorionic gonadotropin.

hCG treatment can promote testosterone synthesis and secretion in LCs [26]. The observation of increased CDK5RAP3 expression from the postnatal stage to sexual maturity led us to hypothesize that this increase might be due to a response to LH/hCG. To test his hypothesis, primary LCs were firstly isolated from male mice approximately 8 weeks of age (Figure S3A); then, the primary LCs and MLTC-1 cells were treated with different hCG concentrations (0, 0.5, 1, and 5 IU/mL) in 6 h, with maximal stimulatory effects at 1 IU/mL (Figure S3B,C). This optimal concentration was subsequently employed for a temporal analysis (0 h, 3 h, 6 h, and 12 h), revealing progressive testosterone production in conditioned media that peaked at 12 h (Figure S3D,E). However, the RT-qPCR and WB results showed that HCG treatment at different time points and concentrations had no significant effect on the *CDK5RAP3* mRNA (Figure S3F–I) and protein expression (Figure 5A–D) in primary LCs and MLTC-1 cells.

To systematically evaluate the potential hCG-mediated regulation of CDK5RAP3 trafficking, primary LCs and MLTC-1 cell lines were subjected to 1 IU/mL hCG treatment for 12 h followed by an immunofluorescence analysis. The results revealed hCG stimulation can induce CDK5RAP3 protein nuclear translocation (Figure 5E,F). This observation was biochemically validated through subcellular fractionation assays in MLTC-1 cells, demonstrating a progressive increase in nuclear CDK5RAP3 protein abundance post-stimulation (Figure 5G).

### 2.6. Effects of CDK5RAP3 on Testosterone Production in Primary LCs and MLTC-1 Cells

**Figure 6:**
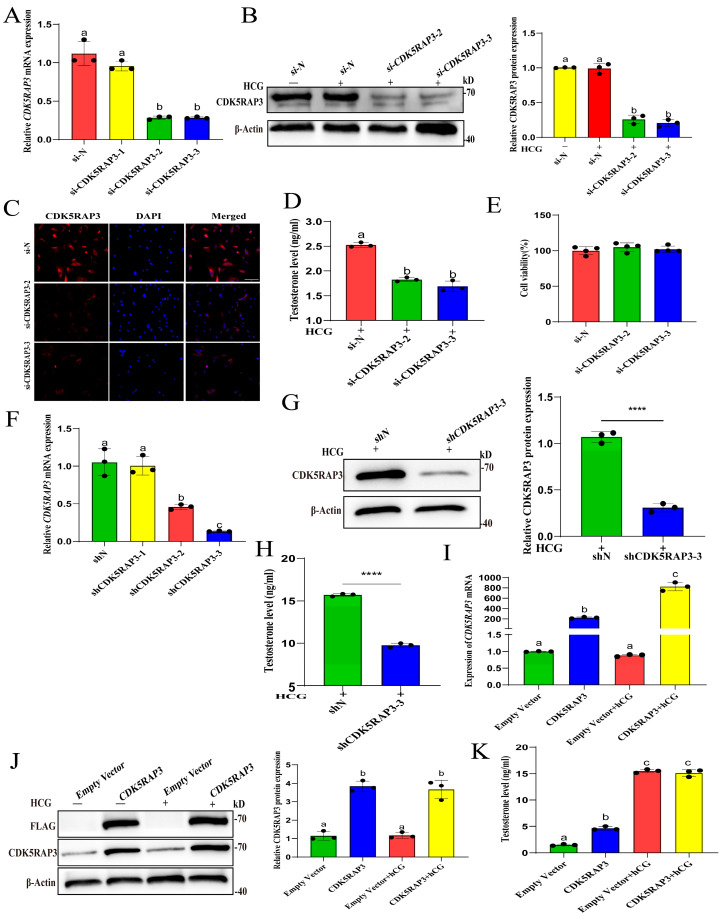
The effect of CDK5RAP3 knockdown/overexpression on testosterone levels in primary LCs and MLTC-1 cells. (**A**) The results of *CDK5RAP3* mRNA after transfection of si-CDK5RAP3 in primary LCs. (**B**) The Western blot results of CDK5RAP3 after transfection of si-CDK5RAP3 in primary LCs. (**C**) Immunofluorescence results of CDK5RAP3 after transfection of si-CDK5RAP3 in primary LCs. Scale bar, 50 µm. (**D**) The results of si-CDK5RAP3 on testosterone synthesis in primary LCs. (**E**) The results of si-CDK5RAP3 on cell viability in primary LCs. (**F**) The results of *CDK5RAP3* mRNA after transfection of shCDK5RAP3 in MLTC-1. (**G**) The Western blot results of CDK5RAP3 after transfection of shCDK5RAP3 in MLTC-1. (**H**) The results of shCDK5RAP3 on testosterone synthesis in MLTC-1. (**I**) The results of *CDK5RAP3* mRNA in MLTC-1 following CDK5RAP3 overexpression. (**J**) The Western blotting results of CDK5RAP3 in MLTC-1 after CDK5RAP3 overexpression. (**K**) The results of CDK5RAP3 overexpression on testosterone synthesis in MLTC-1. Different letters indicate statistically significant differences, *p* < 0.05. **** *p* < 0.0001. Data represent at least three independent biological replicates.

To further verify the role of CDK5RAP3 in hCG-induced testosterone, we utilized siRNA, shRNA lentivirus vector, or pcDNA-CDK5RAP3 to downregulate or upregulate the CDK5RAP3 expression in primary LCs and MLTC-1 cells. As expected, the siRNA or shRNA transfection significantly suppressed the *CDK5RAP3* mRNA and protein expression in primary LCs and MLTC-1 cells, respectively (*p* < 0.05, Figure 6A–C,F,G). After treatment with 1 IU/mL hCG at 6 h, the CDK5RAP3-deficient primary LCs and MLTC-1 cells significantly produced less testosterone compared with the control (*p* < 0.05, Figure 6D,H). However, the CCK-8 assay showed that CDK5RAP3 knockdown did not affect the cell viability of primary LCs (Figure 6E).

To elucidate the regulatory effects of CDK5RAP3 overexpression on basal and hCG-stimulated testosterone synthesis, after treatment with 1 IU/mL hCG at 6 h, conditioned media and cell lysate were collected from four experimental cohorts of MLTC-1 cells: the Empty Vector group, CDK5RAP3 group (CDK5RAP3 overexpression plasmids), hCG + Empty Vector group, and hCG + CDK5RAP3 group. The results showed that, compared with the Empty Vector group, the mRNA and protein expression levels of CDK5RAP3 were significantly increased, transfected with the CDK5RAP3 overexpression plasmid (*p* < 0.05, Figure 6I,J). The testosterone concentration showed that, compared with the Empty Vector group, the testosterone concentration in the CDK5RAP3 group was significantly increased, with statistical significance (*p* < 0.05). However, there was no significant difference in hCG-induced testosterone secretion between CDK5RAP3-overexpressing and control cells (Figure 6K).

### 2.7. Effects of CDK5RAP3 on Steroidogenic Genes in Primary LCs and MLTC-1 Cells

**Figure 7:**
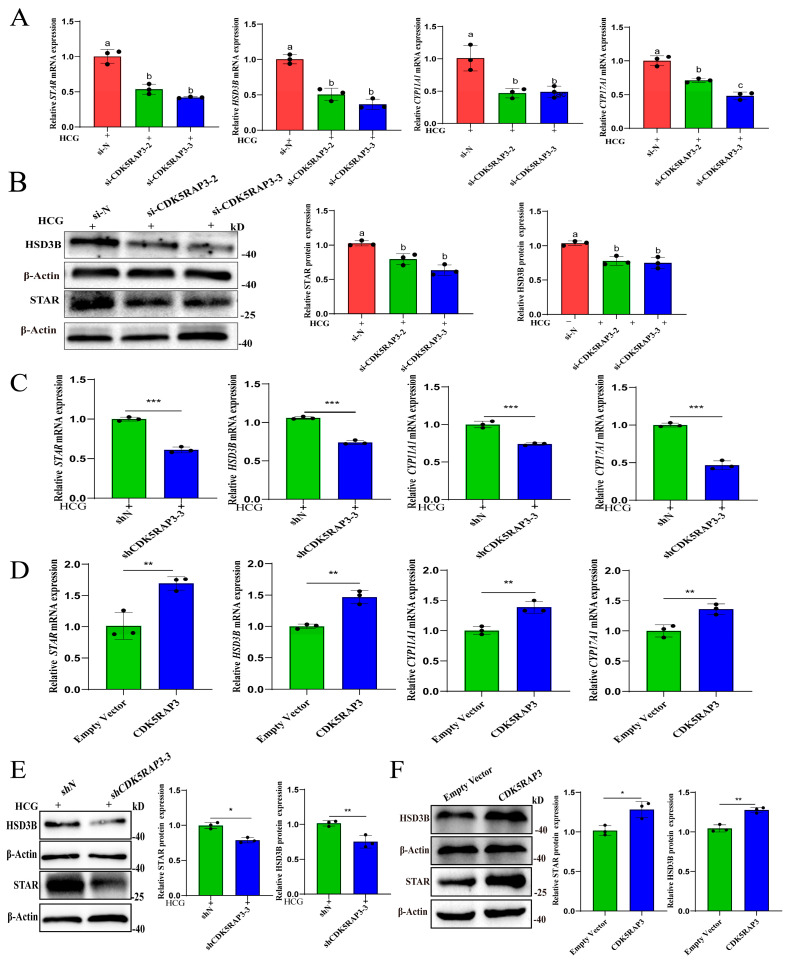
The effect of CDK5RAP3 knockdown/overexpression on steroidogenic gene expression in primary LCs and MLTC-1 cells. (**A**) The mRNA results of steroidogenic genes in si-CDK5RAP3 primary LCs. (**B**) The Western blot results of steroidogenic genes in si-CDK5RAP3 primary LCs. (**C**) The mRNA results of steroidogenic genes in shCDK5RAP3 MLTC-1. (**D**) The mRNA results of steroidogenic genes in CDK5RAP3 overexpression MLTC-1. (**E**) The Western blot results of steroidogenic genes in shCDK5RAP3 MLTC-1. (**F**) The Western blot results of steroidogenic genes in CDK5RAP3 overexpression MLTC-1. Different letters indicate significant differences, *p* < 0.05. * *p* < 0.05. ** *p* < 0.01. *** *p* < 0.001. Data represent at least three independent biological replicates.

To systematically investigate whether CDK5RAP3 participates in testosterone synthesis and secretion by regulating steroidogenic genes in primary LCs and MLTC-1 cells, under 1 IU/mL hCG stimulation for 6 h, we found that CDK5RAP3 knockdown could reduce the mRNA expression of *STAR*, *HSD3B*, *CYP17A1*, and *CYP11A1* (*p* < 0.05, Figure 7A,C), and corresponding protein reductions were observed for STAR and HSD3B (*p* < 0.05, Figure 7B,E).

In overexpression models, CDK5RAP3 overexpression could significantly promote testosterone synthesis in MLTC-1 cells under hCG-free conditions. RT-qPCR and Western blotting were used to assess the mRNA and protein expression levels of steroidogenic genes in CDK5RAP3-overexpressing MLTC-1 cells under hCG-free conditions. The results showed that CDK5RAP3 overexpression could significantly increase the mRNA levels of *STAR*, *HSD3B*, *CYP11A1*, and *CYP17A1* (*p* < 0.05, Figure 7D). At the protein level, consistent upregulation was observed for STAR and HSD3B (*p* < 0.05, Figure 7F). The protein expression of CYP11A1 and CYP17A1 was not evaluated in the overexpression model due to experimental limitations.

### 2.8. CDK5RAP3 Acts Through CEBPB to Regulate Steroidogenic Gene Expression

**Figure 8:**
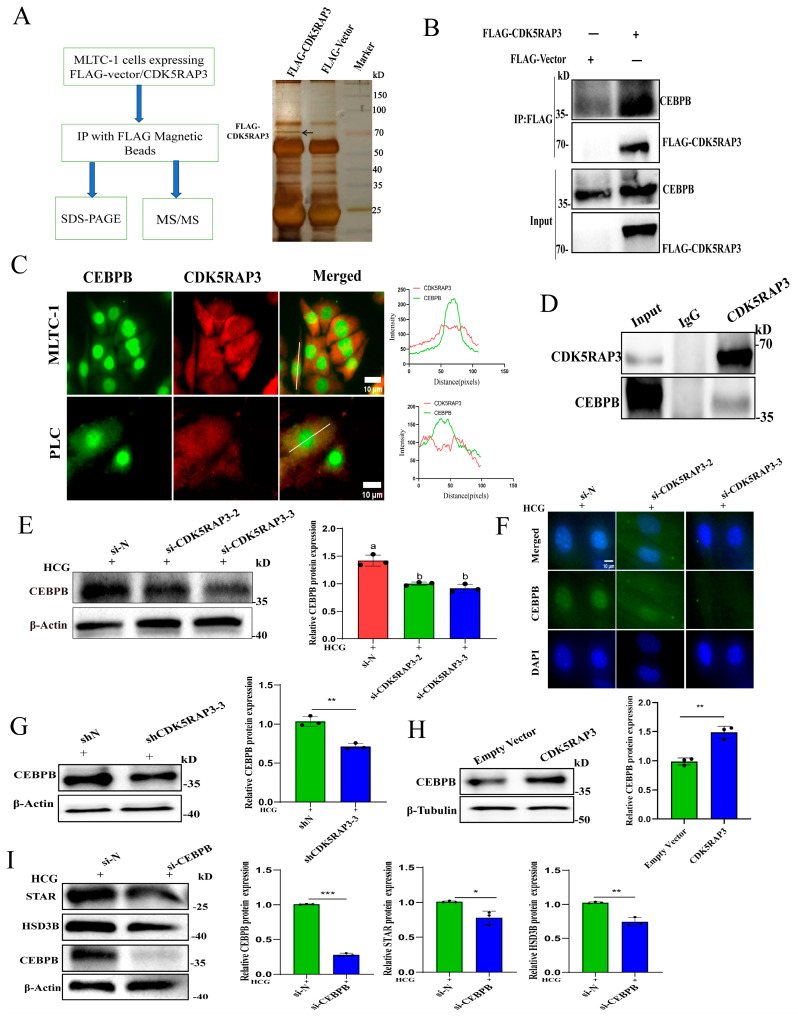
CDK5RAP3 regulate steroidogenic gene expression via interaction with CEBPB. (**A**) The IP-MS results of FLAG-CDK5RAP3 complexes in MLTC-1 cells. (**B**) The Co-IP assay results of CEBPB with FLAG-CDK5RAP3 in MLTC-1 cells. (**C**) Co-localization of CDK5RAP3 and CEBPB in MLTC-1 and primary LCs by immunofluorescence. Scale bar, 10 µm. (**D**) The IP assay results of endogenous CDK5RAP3 and CEBPB in MLTC-1 cells. (**E**) The Western blot results of CEBPB in primary LCs after si-CDK5RAP3 knockdown. (**F**) The immunofluorescence results of CEBPB in primary LCs after si-CDK5RAP3 knockdown. Scale bar, 10 µm. (**G**) The Western blot results of CEBPB after shCDK5RAP3 knockdown in MLTC-1 cells. (**H**) The Western blot results of CEBPB after CDK5RAP3 overexpression in MLTC-1 cells. (**I**) The Western blot results of si-CEBPB Knockdown on steroidogenic gene expression in MLTC-1 Cells. Different letters indicate statistically significant differences, *p* < 0.05. * *p* < 0.05. ** *p* < 0.01. *** *p* < 0.001. Data represent at least three independent biological replicates. Abbreviations: IP-MS, immunoprecipitation followed by mass spectrometry; CEBPB, CCAAT/enhancer-binding protein beta. FLAG-CDK5RAP3 refers to a short epitope tag (DYKDDDDK) fused to pCMV-CDK5RAP3 Vector used for IP-MS or Co-IP assay using anti-FLAG antibody.

In this study, the IP-MS analysis of FLAG-tagged CDK5RAP3 complexes in MLTC-1 cells identified CEBPB as a binding parter. The results confirmed the interaction between CDK5RAP3 and CEBPB (Figure 8A,B). To further confirm the interaction between CDK5RAP3 and CEBPB in primary LCs and MLTC-1 cells, immunofluorescence double staining showed the colocalization of CDK5RAP3 and CEBPB in MLTC-1 and primary LC cells upon 1 IU/mL hCG stimulation at 12 h (Figure 8C), and endogenous IP assays in MLTC-1 cells revealed an interaction between CDK5RAP3 and CEBPB (Figure 8D).

To investigate whether CDK5RAP3 regulates CEBPB expression, CDK5RAP3 knockdown in primary LCs showed a statistically significant reduction in CEBPB expression at both the mRNA and protein levels (*p* < 0.05, Figure 8E,F and Figure S4A). In MLTC-1 cells, CDK5RAP3 knockdown or overexpression led to a significant decrease or increase in CEBPB expression at both the mRNA and protein levels, respectively (*p* < 0.05, Figure 8F,G and Figure S4B). Meanwhile, under 1 IU/mL hCG stimulation for 6 h, CEBPB knockdown could reduce the mRNA expression of *STAR*, *HSD3B*, and *CYP17A1* (*p* < 0.05, Figure S4C), and corresponding protein reductions were observed for STAR (*p* < 0.05, Figure 8I).

### 2.9. CDK5RAP3 Regulates Testosterone Synthesis Through SMAD4/CEBPB Axis

**Figure 9:**
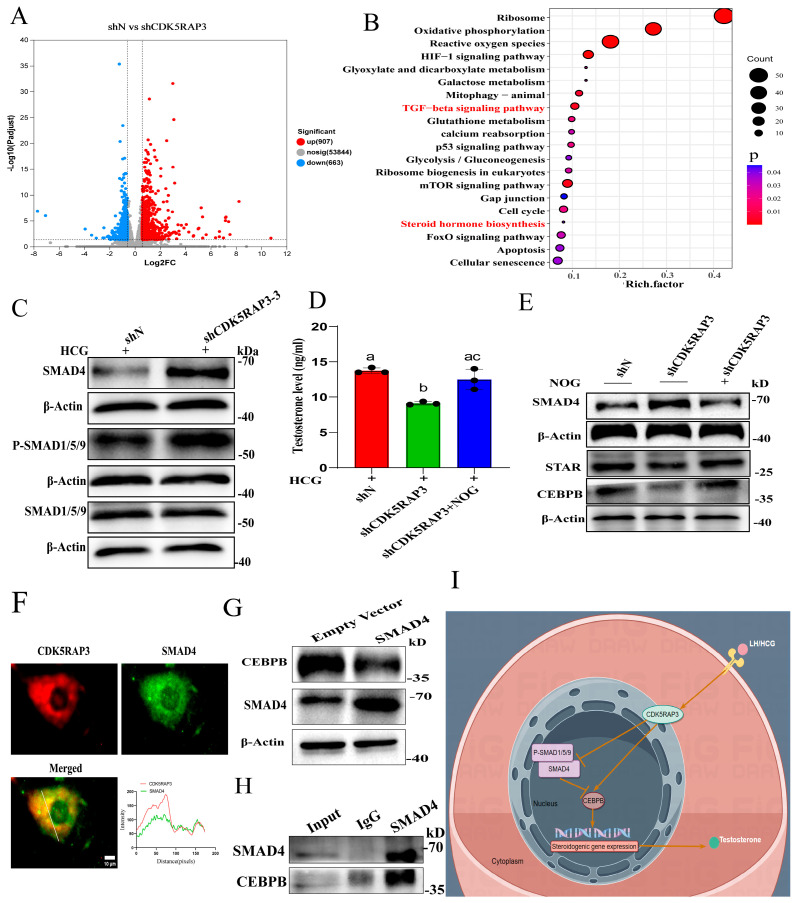
CDK5RAP3 regulates testosterone secretion through SMAD4/CEBPB axis. (**A**) Volcano plot showing differentially expressed genes between shN and shCDK5RAP3 MLTC-1 cells (*n* = 3 per group). (**B**) KEGG enrichment analysis results of differentially expressed genes. (**C**) The Western blot results of SMAD4 and P-SMAD1/5/9 in shCDK5RAP3 MLTC-1 cells. (**D**) ELISA results of Noggin reversing testosterone secretion inhibited by shCDK5RAP3. (**E**) The Western blot results of SMAD4, CEBPB, and STAR in shCDK5RAP3 MLTC-1 cells treated with NOG. (**F**) Immunofluorescence co-localization of CDK5RAP3 and SMAD4 in primary LC cells. (**G**) The Western blot results of SMAD4 and CEBPB in SMAD4 overexpression MLTC-1cells. (**H**) The IP assay results of endogenous SMAD4 and CEBPB in MLTC-1 cells. (**I**) Schematic diagram of CDK5RAP3 regulating testosterone secretion pathways in Leydig cells. Different letters indicate statistically significant differences, *p* < 0.05. Data represent at least three independent biological replicates. Abbreviations: STAR, steroidogenic acute regulatory protein; NOG, Noggin.

Following 6 h of hCG stimulation, transcriptomic profiling revealed 1579 differentially expressed genes (DEGs) between shCDK5RAP3 and shN MLTC-1 cells, including 907 upregulated and 663 downregulated genes (Figure 9A). A KEGG pathway analysis showed the significant enrichment of pathways including HIF-1, mTOR, FOXO, and TGF-β signaling (Figure 9B) [27,28,29,30]. A further analysis of DEGs within the TGF-β pathway identified BMP4, ID1, and ID3 as prominently enriched genes (Figure S5A).

We firstly assessed SMAD4 and phospho-SMAD1/5/9 protein levels in shN and shCDK5RAP3 MLTC-1 cells, all which were significantly elevated in shCDK5RAP3 cells compared to shN controls (*p* < 0.05, Figure 9C and Figure S5B). Additionally, CDK5RAP3 overexpression also inhibited the protein expression levels of SMAD4 (*p* < 0.05, Figure S5C). To directly test whether CDK5RAP3 influences testosterone synthesis via the BMP/SMAD pathway, shCDK5RAP3 MLTC-1 cells were treated with the BMP antagonist Noggin for 24 h, followed by hCG stimulation for 6 h. Testosterone levels in the supernatant and quantitative analysis of related proteins were then analyzed by ELISA and Western blotting, respectively. Noggin treatment significantly increased the testosterone concentration in shCDK5RAP3 MLTC-1 cells compared to untreated shCDK5RAP3 cells (*p* < 0.05, Figure 9D). Concurrently, a Western blot analysis demonstrated that Noggin treatment significantly upregulated the protein expression of SMAD4, CEBPB, and STAR in shCDK5RAP3 MLTC-1 cells (*p* < 0.05, Figure 9E and Figure S5D).

A BioGRID database analysis predicted an interaction between CDK5RAP3 and SMAD4; to confirm the interaction between CDK5RAP3 and SMAD4 in primary LCs, immunofluorescence double staining showed the colocalization of CDK5RAP3 and SMAD4 in primary LC cells (Figure 9F). To investigate this relationship in our model, MLTC-1 cells were transfected with a SMAD4 overexpression plasmid. Western blotting revealed that SMAD4 overexpression significantly increased SMAD4 protein levels while concurrently suppressing CEBPB expression compared to empty vector controls (*p* < 0.05, Figure 9G and Figure S5E), indicating SMAD4 inhibits CEBPB synthesis in MLTC-1 cells. Endogenous IP was performed using SMAD4 antibody in MLTC-1 cell lysates, and Western blotting confirmed the endogenous interaction between SMAD4 and CEBPB (Figure 9H). Collectively, these findings suggest CDK5RAP3 may regulate Leydig cell testosterone synthesis via a BMP/SMAD/CEBPB interaction axis (Figure 9I).

## 3. Discussion

Previous studies have demonstrated that CDK5RAP3 is widely distributed across tissues, with reported expression in various organ systems such as the liver [31,32], intestinal Paneth cells [33], and the central nervous system [19], where it functions as a critical molecule in maintaining normal cell cycle progression and tissue development.

In the present study, we expanded these findings to the male reproductive system. The widespread expression of CDK5RAP3 was observed in both human and mouse testes, and IP-MS analysis for CDK5RAP3 interacting proteins in mouse testicular tissue identified CDK5RAP3 as a hub protein associated with steroidogenic- and cholesterol-metabolism-related networks. Since the synthesis and secretion of steroid hormones are mainly mediated by LCs in the testes [34], these findings suggest a potential link between CDK5RAP3 and Leydig cell function.

Leydig cell dysfunction is known to cause male reproductive disorders, including testosterone deficiency, hypogonadism, and impaired spermatogenesis [35]. Immunofluorescence analysis showed a reduced CDK5RAP3 expression in Leydig cells from patients with low serum testosterone, and CDK5RAP3 levels were positively correlated with HSD3B expression, a key rate-limiting enzyme in testosterone biosynthesis [36,37].

In mammals, LCs progressively acquire the capacity to synthesize testosterone as they differentiate from stem LCs to ALCs, reaching peak steroidogenic activity in the ALCs [38,39]. In mice, both *CDK5RAP3* mRNA and protein expression levels exhibited an age-dependent increase. Notably, the CDK5RAP3 expression was higher in ALCs than in PLCs. The differentiation and function of LCs rely on finely regulated gene expression programs [40,41]. In the rat EDS-induced Leydig cell regeneration model, the CDK5RAP3 expression increased progressively during Leydig cell regeneration, following a trend similar to that of key steroidogenic genes such as STAR and HSD3B. Based on these observations, we propose that CDK5RAP3 may be involved in the development of testicular LCs and in the regulation of testosterone biosynthesis and secretion.

In primary LCs and MLTC-1 cells, hCG treatment led to a significant increase in testosterone levels without altering the *CDK5RAP3* mRNA and protein expression. Given that CDK5RAP3 has been described as a dynamic nucleocytoplasmic shuttle protein exhibiting stimulus-dependent subcellular redistribution [42], IF and nuclear-cytoplasmic fractionation experiments revealed CDK5RAP3 underwent nuclear translocation following treatment with 1 IU/mL hCG for 12 h, suggesting its involvement in testosterone production. In LCs, cholesterol is converted to testosterone by key steroidogenic genes, including STAR, HSD3B, CYP11A1, and CYP17A1 [43]. Subsequent knockdown experiments demonstrated that the depletion of CDK5RAP3 significantly reduced both testosterone production and the expression of steroidogenic genes in both primary LCs and MLTC-1 cells. Conversely, CDK5RAP3 overexpression in MLTC-1 cells without hCG stimulation significantly enhanced testosterone levels and increased the expression of steroidogenic genes. However, after 6 h of 1 IU/mL hCG stimulation, no statistically significant difference in testosterone secretion was observed between CDK5RAP3-overexpressing cells and vector controls. This finding is supported by the experimental observation that hCG can alone robustly activate steroidogenic pathways in MLTC-1 cells. It is therefore plausible that hCG stimulation induces a near-maximal steroidogenic response, thereby limiting the additional impact of CDK5RAP3 overexpression under these conditions.

The in vivo knockdown of CDK5RAP3 in LCs using AAV2/9-shCDK5RAP3 further confirmed its regulatory role in testosterone production and its influence on apoptosis within the seminiferous tubules. EGFP fluorescence revealed that AAV2/9 particles predominantly infected testicular LCs, with no detectable EGFP expression in the seminiferous tubules, consistent with previous findings [44,45]. CDK5RAP3 downregulation significantly reduced the serum testosterone levels in mice, while the testicular weight and sperm count remained unaffected. The TUNEL assay revealed a statistically significant increase in apoptotic cells within the seminiferous tubules of the AAV2/9-shCDK5RAP3 group. No apoptotic events were detected in LCs, suggesting that CDK5RAP3 specifically regulates testosterone synthesis and secretion in these cells without directly affecting apoptosis. The reduction in CDK5RAP3 expression in LCs led to decreased testosterone levels, which may have secondarily compromised the survival of Sertoli or spermatogenic cells, contributing to increased apoptosis. However, spermatogenesis in mice is a prolonged process requiring several complete cycles for sperm maturation [46]. Although AAV2/9-shCDK5RAP3 effectively suppressed CDK5RAP3 in LCs, this was insufficient to induce a rapid decline in sperm count. Thus, CDK5RAP3 downregulation primarily impairs Leydig cell steroidogenic function, resulting in decreased serum testosterone levels. Its effect on germ cell survival likely operates through a delayed, indirect mechanism, with no significant direct impact on testicular weight or sperm output. These findings collectively underscore the essential role of CDK5RAP3 in regulating testosterone synthesis at both the cellular and tissue levels.

CDK5RAP3 contains a leucine zipper domain that facilitates protein dimerization and two LXXLL motifs that mediate interactions with transcription factors, suggesting its potential role as a transcriptional regulator [16]. The identification of CEBPB as a CDK5RAP3-interacting partner via IP-MS represents a significant discovery, considering CEBPB’s established role as a transcription factor in steroidogenesis and Leydig cell development [8,10]. This interaction was robustly validated through complementary approaches including co-immunoprecipitation and immunofluorescence. Bidirectional modulation experiments (both knockdown and overexpression) in primary LCs and MLTC-1 cells provided particularly informative results. The observed correlation between CDK5RAP3 and CEBPB expression at both the mRNA and protein levels suggests that CDK5RAP3 may regulate CEBPB via multiple mechanisms: (1) the transcriptional regulation of CEBPB expression; (2) the post-translational stabilization of CEBPB protein; and (3) the modulation of its interaction with transcriptional co-regulators.

The TGF-β pathway has been specifically linked to steroidogenesis, Leydig cell differentiation, and testosterone synthesis [14,47,48]. As TGF-β signaling encompasses ligands such as TGF-β1/2/3 and BMPs (bone morphogenetic proteins) [49], the transcriptomic analysis and Western blotting of shCDK5RAP3 and shN-transfected MLTC-1 cells demonstrated that CDK5RAP3 regulates testosterone synthesis through the BMP/SMAD signaling pathway. CDK5RAP3 knockdown significantly upregulated SMAD4 and phosphorylated SMAD1/5/9 proteins. Moreover, treatment with the BMP inhibitor Noggin reversed the reduction in testosterone production and steroidogenic gene expression caused by CDK5RAP3 knockdown.

The BioGRID database analysis predicted a potential interaction between CDK5RAP3 and SMAD4. However, mass spectrometry did not detect this interaction; instead, it revealed an interaction between CDK5RAP3 and SMAD7, a known inhibitor of SMAD4. It is speculated that CDK5RAP3 overexpression may suppress SMAD4 expression, thereby disrupting the CDK5RAP3–SMAD4 interaction and preventing its detection by mass spectrometry. To resolve this discrepancy, immunofluorescence experiments confirmed a direct interaction between CDK5RAP3 and SMAD4. These findings indicate that CDK5RAP3 likely modulates the BMP/SMAD signaling cascade—and, hence, testosterone production—via the interaction with SMAD4.

Noggin treatment also reversed the CDK5RAP3-knockdown-induced suppression of CEBPB, suggesting that the BMP/SMAD pathway may regulate testosterone production through the modulation of CEBPB. This is supported by previous reports that SMAD4 inhibits CEBPB-driven transcription [50] and interacts with CEBPB to promote lipid droplet formation in bovine myoblasts [51]. The BioGRID analysis also predicted an interaction between SMAD4 and CEBPB. To validate this interaction, the transfection of an SMAD4 overexpression plasmid into MLTC-1 cells confirmed that SMAD4 downregulates CEBPB expression. Endogenous co-immunoprecipitation further confirmed the interaction between SMAD4 and CEBPB.

Nevertheless, several limitations of the present study should be acknowledged. The relatively small sample size of human specimens and animal cohorts may restrict the generalizability of these findings. Further investigations in larger and independent cohorts will be necessary.

In summary, our findings demonstrate that CDK5RAP3 regulates testicular testosterone synthesis by forming a regulatory complex with SMAD4 and CEBPB, thereby modulating the expression of key steroidogenic genes.

## 4. Materials and Methods

### 4.1. Antibodies and Reagents

The following antibodies were used in this study: Anti-CDK5RAP3 (Cat#: Ab157203) from Abcam (Cambridge, MA, USA); Anti-CEBPB (Cat#: sc-7962) and Anti-SMAD4 (Cat#: sc-7966, used for IF) from Santa Cruz Biotechnology (Dallas, TX, USA); Anti-STAR (Cat#: 80751-1-RR), Anti-GAPDH (Cat#: 60004), Anti-SMAD4 (Cat#: 10231-1-AP, used for IP), Anti-FLAG (Cat#: 20543-1-AP), Anti-HA (Cat#: 51064-2-AP), Anti-β-Actin (Cat#: 7D2C10), and Anti-Lamin B1 (Cat#: 12987) from Proteintech (Wuhan, China); Anti-HSD3B (Cat#: DF6639) and Anti-SMAD1/5/9 (Cat#: AF0614) from Affinity (Changzhou, China); Anti-P-SMAD1/5/9 (Cat#: 13820) from CST (Beverley, MA, USA); CY3-conjugated goat anti-rabbit (Cat#: BA1032) and FITC-conjugated goat anti-mouse (Cat#: BA1101) from BOSTER (Wuhan, China); and HRP-Conjugated Goat Anti-Mouse IgG (Cat#: BL001A) and HRP-Conjugated Goat Anti-Rabbit IgG (Cat#: BL003A) from Biosharp (Hefei, China).

The following reagents were used: Noggin (Cat#: HY-P7086) and EDS (Cat#: HY-129524) from MCE (Shanghai, China); hCG (Cat#: H20052130) from Livzon (Zhuhai, China); RNAiMAX (Cat#: 13778075), Lip3000 (Cat#: L3000015), and Pierce™ Protein A/G Magnetic Beads (Cat#: 88802) from Thermo Scientific (Waltham, MA, USA); collagenase type IV (Cat#: BS165) from Biosharp (Hefei, China); Testosterone Chemiluminescence Kit (Cat#: K-1408-100N) from Kangrun (Guangzhou, China); ELISA Kit (Cat#: HLE30439) from Haling (Shanghai, China); CCK8 kit (Cat#: C0042), TUNEL Apoptosis Assay Kit (Cat#: C1089), RIPA lysis buffer (Cat#: P0013B), and Nuclear and Cytoplasmic Separation Kit (Cat#: P0027) from Beyotime (Shanghai, China).

### 4.2. Animal Experiments and Study Participants

Kunming (KM) mice and Sprague–Dawley (SD) rats were purchased from Changsha Tianqin Biotechnology Company. KM mice were used for the isolation of primary Leydig cells and AAV injection experiments in the testes in vivo, while SD rats were utilized for the establishment of EDS-induced Leydig cell regeneration model. The animals were housed under a 12 h light/dark cycle at a controlled temperature (25 ± 1 °C) and relative humidity of 50–60%, with free access to feed and drinking water. Animals were anesthetized with sodium pentobarbital (50 mg/kg) through intraperitoneal injection prior to all invasive procedures to minimize pain and distress. Adequate depth of anesthesia was confirmed by the absence of pedal withdrawal reflex, and animals were continuously monitored throughout the procedures. All procedures were performed under aseptic conditions following institutional animal care guidelines.

Testicular tissue sections of OA patients (Controls, T > 10.4 nmol/L, *n* = 4) and spermatogenic failure patients (T < 10.4 nmol/L, *n* = 6) were obtained from the Department of Pathology of the First Affiliated Hospital of Wannan Medical College. All experiments involving these mice were conducted with the approval of the Institutional Animal Care and Use Committee of Anhui Normal University.

### 4.3. LC Isolation and Culture

In rodents, fibroblast-like PLCs emerge in the testis between postnatal days 14 and 21, whereas ALCs develop between postnatal days 49 and 56. Based on this timeline, PLCs were obtained from 3-week-old male KM mice, while ALCs were isolated from 8-week-old male KM mice. Briefly, decapsulated testes were enzymatically digested with 1 mg/mL collagenase type IV at 37 °C in a shaking water bath for 7 min [52]. The resulting cell suspension was cultured in DMEM/F12 supplemented with 10% FBS for 1 h, after which the medium was replaced to eliminate non-adherent blood cells, spermatocytes, and Sertoli cells. The remaining adherent cells were then incubated at 37 °C with 5% CO_2_. To further purify Leydig cells, a 2 min hypotonic treatment (20 mM Tris, pH 7.4) was applied after 24 h to remove myoid cells. The purified cells were maintained in culture for an additional 48 h before being used for subsequent experiments.

### 4.4. EDS-Induced Leydig Cell Regeneration in Rat Models

A Leydig cell regeneration model was established in adult SD rats using EDS-induced ablation, as previously described [53]. Rats (*n* = 4 per group) received a single intraperitoneal injection of EDS (75 mg/kg body weight), a dose optimized to induce specific and complete elimination of testicular LCs. Regenerating Leydig cell populations were subsequently classified based on established morphological characteristics and the expression of well-recognized steroidogenic markers, as described in previous studies [54]. Histological analyses confirmed complete depletion of LCs by PTD7. By PTD14, HSD3B-positive cells with distinct morphological characteristics became detectable, consistent with the emergence of regenerating progenitor Leydig cells RPLCs. At PTD28, round cells containing abundant lipid droplets and displaying structural and functional features of immature LCs were observed, termed RILCs [55].

### 4.5. siRNA/shRNA Knockdown

siRNAs were designed and synthesized by Generalbiol, China. The sequences of the siRNAs are listed: si-CDK5RAP3-1 (5′-GAAACUGAUUGAAGCCGACAUTT-3′), si-CDK5RAP3-2 (5′-CCAGAGAGCUGCAGAAACUTT-3′), si-CDK5RAP3-3 (5′-UCAUGGAGCUUGAGAUCUUTT-3′), and si-CEBPB (5′-CACCCUGCGGAACUUGUUCAATT-3′). Cells were transfected with si-CDK5RAP3/CEBPB or scrambled siRNA (negative control) using Lipofectamine RNAiMAX reagent according to the manufacturer’s protocol. After 48 h, knockdown efficiency was assessed by RT-qPCR and Western blotting. To evaluate functional effects, cells were treated with hCG stimulation for 6 h, and testosterone levels in the culture medium were quantified by ELISA. Additionally, gene and protein expression of key steroidogenic genes were analyzed.

The lentiviral vector VP018-U6-MCS-PGK-PURO encoding CDK5RAP3 shRNAs (sh-CDK5RAP3) and a nonsilencing negative control (shN) were constructed by Generalbiol, China. The shCDK5RAP3 target sequences were as follows: shCDK5RAP3-1 (CCAGCAACTGCAGCAAGAATA), shCDK5RAP3-2 (GCTGAGATGCGAGAGCAATTC), and shCDK5RAP3-3 (GCTCTGACTCTTCTGGAATAC). MLTC-1 cells were seeded at a density of 2 × 10^5^ cells/well in six-well plates and transduced with lentiviral particles at a multiplicity of infection (MOI) of 20 in the presence of 16 μg/mL polybrene to enhance infection efficiency as previously described [56]. Following overnight incubation, the viral medium was replaced with fresh culture medium, and the cells were maintained for an additional 48 h. Subsequently, cells and culture supernatants were collected after treatment with 1 IU/mL hCG for 6 h.

### 4.6. Plasmid Transfection in MLTC-1 Cells

Two experimental plasmid constructs were employed: a pCMV-CDK5RAP3 vector encoding full-length mouse CDK5RAP3 cDNA under control of the CMV promoter fused with an N-terminal FLAG epitope tag (FLAG-CDK5RAP3), and pCMV-Null empty vector serving as negative control. The FLAG tag (DYKDDDDK) is a short epitope sequence fused to CDK5RAP3 to facilitate protein detection and immunoprecipitation using anti-FLAG antibodies.

MLTC-1 cells were transfected at 70–80% confluence. Transfection was performed using Lipofectamine 3000 reagent (Thermo Scientific, Waltham, MA, USA) according to the manufacturer’s protocol. FLAG-CDK5RAP3-transfected cells were used for overexpression, immunoprecipitation, and downstream functional assays.

### 4.7. RNA Sequencing and Bioinformatic Analysis

Total RNA was extracted from shCDK5RAP3 and shN MLTC-1 cells (*n* = 3 per group) after 6 h of hCG stimulation using TRIzol reagent (Invitrogen, Carlsbad, CA, USA), and RNA integrity was assessed prior to library construction. RNA-seq libraries were prepared using a standard poly (A) enrichment protocol and sequenced on Majorbio Company. Raw reads were quality-filtered and aligned to the mouse reference genome (GRCm38) using HISAT2, and gene expression levels were quantified as fragments per kilobase of transcript per million mapped reads (FPKM). Differentially expressed genes were identified using DESeq2 (version 1.40.2) with an adjusted *p* < 0.05 and |log_2_FC| > 1. Functional enrichment analyses were performed using Gene Ontology and KEGG pathway databases.

### 4.8. AAV2/9-ShCDK5RAP3-Mediated CDK5RAP3 Knockdown in the Testes

To investigate the role of CDK5RAP3 in testosterone synthesis and secretion in vivo, 20 µL of AAV2/9-shCDK5RAP3 (OBiO Technology, Shanghai, China) at a concentration of 5 × 10^12^ v.g./mL was injected into the testes of male KM mice. Eight-week-old male mice were randomly divided into two groups, AAV2/9-shRNA control and AAV2/9-shCDK5RAP3 (GCTCTGACTCTTCTGGAATAC), with six mice per group. Under sterile conditions, the testes were surgically exposed via an abdominal approach, and either AAV2/9-shRNA or AAV2/9-shCDK5RAP3 was injected directly into the testes as previously described [25]. The viral suspension contained 0.04% trypan blue, which ensured uniform distribution of the virus within the testicular tissue, as indicated by a blue coloration. After 30 days, serum was collected, and both testes and epididymides were harvested for further analysis.

### 4.9. Testosterone Measurement

KM mice and SD rats were anesthetized, and blood was collected via enucleation. The blood samples were allowed to stand at room temperature for 30 min and then centrifuged at 5000× *g* for 5 min to separate the serum. Serum testosterone levels were measured using the Kaeser 6600 (Kangrun Biotech, Guangzhou, China) fully automated chemiluminescence immunoassay analyzer according to the manufacturer’s protocol.

Cell culture supernatants were collected into centrifuge tubes and centrifuged at 3000× *g* for 20 min to remove cell debris and impurities. The testosterone concentration in the culture medium was then measured using an ELISA assay (Haling, Shanhghai, China) following the manufacturer’s instructions. The minimum detectable concentration of testosterone was 0.05 ng/mL. The intra- and inter-assay coefficients of variation of the assay were <10% and 15%, respectively.

### 4.10. RNA Extraction, cDNA, and Quantitative Real-Time PCR

Testes, primary LCs, and MLTC-1 cells were dissolved with Trizol reagent (Cat#: 15596026CN, Invitrogen, Carlsbad, CA, USA). RNA purity and concentration were measured using a multifunctional microplate reader (TECAN SPARK, Tecan AG, Männedorf, Switzerland). The absorbance ratios (260/280 nm) of all samples ranged from 1.8 to 2.0. cDNA was synthesized using the KR103 kit (Tiangen Biochemical Technology, Beijing, China). The qPCR primers are listed in Table S1. qRT-PCR was performed on the LightCycler96 detection system (Roche, Basel, Switzerland) using the KR123 kit (Tiangen Biochemical Technology, Beijing, China). Each sample was analyzed in triplicate as technical replicates. Relative gene expression levels were calculated using the 2^−ΔΔCt^ method [57].

### 4.11. Immunoprecipitation Assay

Tissues or cells were lysed in RIPA lysis buffer (Beyotime, Shanghai, China) supplemented with protease inhibitors (Sellect, Dallas, TX, USA). The lysates were centrifuged, and then the supernatant were used for immunoprecipitation, which was carried out by incubating with relevant antibody at 4 °C overnight, followed by incubation with Pierce Protein A/G Magnetic Beads (Thermo Fisher Scientific, Waltham, MA, USA) for 1 h to bind the antibody mixture according to the manufacturer’s protocol. The magnetic beads were then washed three times with IP buffer and once with purified water. After elution, the immunoprecipitates were boiled for 5 min at 95 °C in SDS loading buffer.

### 4.12. Histological Analysis

Testis samples were fixed in animal testicular tissue fixative and embedded in paraffin following standard procedures. Subsequently, 5 μm-thick tissue sections were prepared and subjected to hematoxylin and eosin (H&E) or immunohistochemistry staining according to standard protocols as previously described [58,59]. Finally, bright-field images were captured using a Leica DMi8 microscope (Leica, Wetzlar, Germany). The antibodies used were as follows: Anti-CDK5RAP3 (1:100 dilution) and Anti-HSD3B (1:100 dilution).

### 4.13. Immunocytofluorescence Staining

The cells were fixed for 15 min in 4% paraformaldehyde (PFA) and then permeabilized with 0.1% Triton X-100, and then blocked with BDT (containing 10% normal goat serum in PBST) for 0.5 h at room temperature. The cells were incubated with primary antibodies overnight at 4 °C. The cells were then incubated with fluorescent secondary antibodies for 1.5 h and stained with DAPI for 5 min. A Leica DMi8 fluorescence microscope (Leica, Wetzlar, Germany) was used for observation and photography. The antibodies used were as follows: Anti-CDK5RAP3 (1:100 dilution), Anti-CEBPB (1:50 dilution), Anti-SMAD4 (1:50 dilution), CY3-conjugated goat anti-rabbit (1:100 dilution), and FITC-conjugated goat anti-mouse (1:100 dilution).

### 4.14. Western Blots

Total protein was extracted using RIPA lysis buffer (Beyotime, Shanghai, China) supplemented with protease inhibitors (Sellect) and adjusted to the same concentration (1 µg/µL) with a BCA protein assay kit (Beyotime, Shanghai, China). SDS-PAGE was used to separate proteins, which were then transferred onto PVDF (Millipore, Burlington, MA, USA) membranes [60]. The membranes were then blocked with 5% BSA at room temperature for 1 h and incubated with primary antibodies overnight at 4 °C, followed by a corresponding HRP-conjugated secondary antibody at room temperature for 1.5 h, and then reacted with chemiluminescent substrates (Beyotime, Shanghai, China). The signals were observed using a Tanon 5200 imaging system (Tanon, Shanghai, China). The antibodies used were as follows: Anti-CDK5RAP3 (1:1000 dilution), Anti-STAR (1:1000 dilution), Anti-HSD3B (1:1000 dilution), Anti-CEBPB (1:500 dilution), Anti-SMAD4 (1:1000 dilution), Anti-SMAD1/5/9 (1:1000 dilution), Anti-P-SMAD1/5/9 (1:1000 dilution), Anti-β-Actin (1:1000 dilution), Anti-GAPDH (1:1000 dilution), Anti-Lamin B1 (1:1000 dilution), HRP-Conjugated Goat Anti-Mouse IgG (1:2000 dilution), HRP-Conjugated Goat Anti-Rabbit IgG (1:2000 dilution), Anti-FLAG (1:1000 dilution), and Anti-HA (1:1000 dilution).

### 4.15. TUNEL Staining

Cell apoptosis in the testicular tissue was detected by TUNEL staining (Beyotime, Shanghai, China) according to the manufacturer’s protocol. Briefly, testis sections were incubated in 20 ug/mL Protease K (37 °C, 15 min) and then TUNEL reaction mixture (37 °C, 60 min). Cell nuclei were stained with DAPI. The images were captured by a Leica DMi8 fluorescence microscope (Leica, Wetzlar, Germany).

### 4.16. Statistical Analysis

The data are presented as mean ± SEM. Statistical analysis was performed using GraphPad Prism 8.0 software. The non-parametric *t*-test was used for comparison between two groups, and Kruskal–Wallis test was used for multiple groups of data. The symbol “*” or different lowercase letters indicate significant differences, “*” represents *p* < 0.05, and the letter sequence starts from the left.

## 5. Conclusions

This study indicates that CDK5RAP3 is closely associated with steroidogenesis and cholesterol metabolism, and that its expression is reduced in Leydig cells from patients with low-testosterone-associated spermatogenic disorders. Experimental evidence from mouse testicular development, and PLC and ALC isolation, as well as a rat model of EDS-induced Leydig cell ablation and regeneration, demonstrates that CDK5RAP3 expression progressively increases during the development of testicular Leydig cells. In both primary LC and MLTC-1 cells, hCG stimulation induces the nuclear translocation of CDK5RAP3 protein from the cytoplasm. CDK5RAP3 knockdown markedly reduces testosterone levels and the expression of key steroidogenic genes in both primary LCs and MLTC-1 cells. Conversely, the overexpression of CDK5RAP3 in MLTC-1 cells significantly enhances testosterone production and steroidogenic gene expression in the absence of hCG stimulation. The in vivo knockdown of CDK5RAP3 in mouse testicular Leydig cells via AAV2/9-shCDK5RAP3 further confirmed that CDK5RAP3 is proposed to modulate testosterone secretion and affects germ cell apoptosis within seminiferous tubules. Mechanistically, CDK5RAP3 forms a functional complex with SMAD4 and CEBPB, as an important component of the regulatory network governing testosterone production in testicular LCs. These findings provide new insights into the expression pattern and physiological function of CDK5RAP3 in LCs.

## Data Availability

The data presented in this study are available on request from the corresponding authors.

## Supplementary Materials

The following supporting information can be downloaded at: https://www.mdpi.com/article/10.3390/ijms27020586/s1.

## Abbreviations

The following abbreviations are used in this manuscript:

CDK5RAP3	CDK5 regulatory subunit-associated protein 3
LXXLL	Leucine-X-Leucine-Leucine-Leucine
EDS	ethane dimethanesulfonate
IP-MS	Immunoprecipitation-Mass
RILCs	regenerating immature Leydig cells
RPLCs	generating progenitor Leydig cells
STAR	steroidogenic acute regulatory protein
CYP11A1	Linear dichroism cytochrome P450 family 11 subfamily A member 1
HSD3B	3β-hydroxysteroid dehydrogenase
hCG	human chorionic gonadotropin
LCs	Leydig cells
NOA	non-obstructive azoospermia
NOG	Noggin
T	Testosterone
