# Supplementary material for: CDK5RAP3 Regulates Testosterone Production in Mouse Leydig Cells
Source: Int J Mol Sci. 2026 Jan 6;27(2):586. doi: 10.3390/ijms27020586 (PMC12840814; doi:10.3390/ijms27020586)
Supplement: Supplementary file 1 [file ijms-27-00586-s001.zip › ijms-4038101-supplementary.pdf]

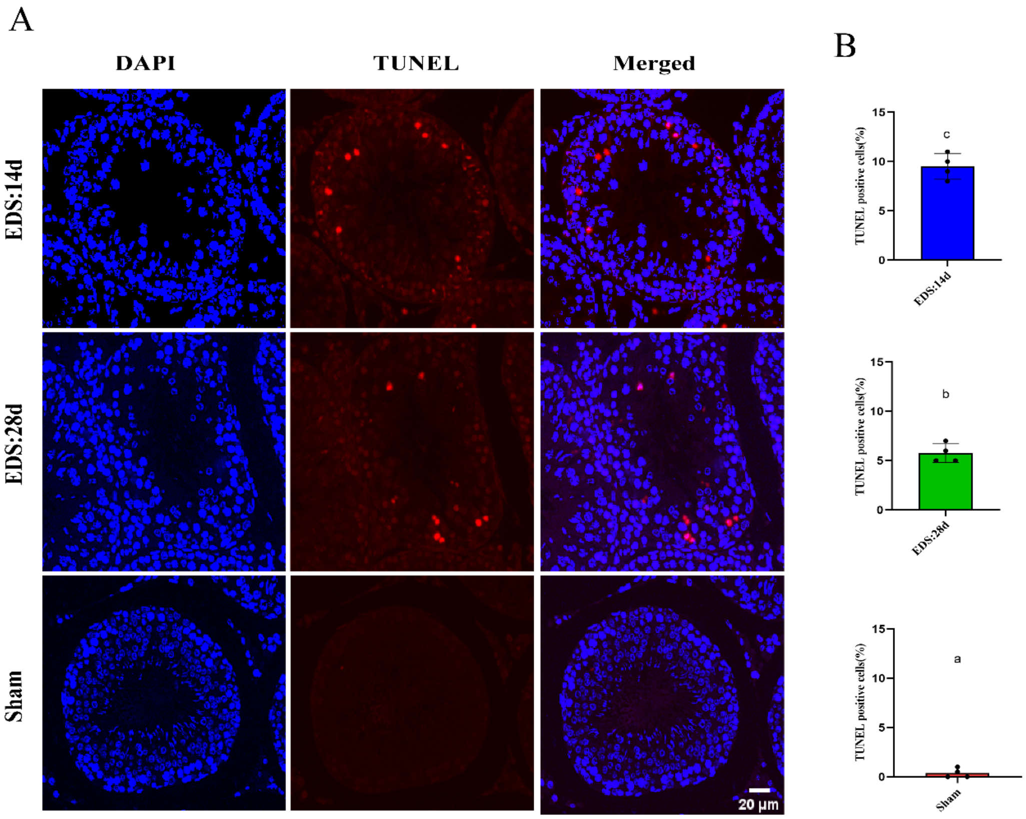

Figure S1. TUNEL Assay Results of Testicular Tissues in the Rat EDS-Induced Model. (n=4 per group) (A) TUNEL assay of testicular tissues from the rat EDS-induced model; (B) Quantitative analysis of TUNEL-positive cells. Different letters indicate significant differences, $P < 0.05$. Scale bar, 20 μm. Abbreviations: EDS, ethane dimethanesulfonate; TUNEL, terminal deoxynucleotidyl transferase–mediated dUTP nick end labeling.

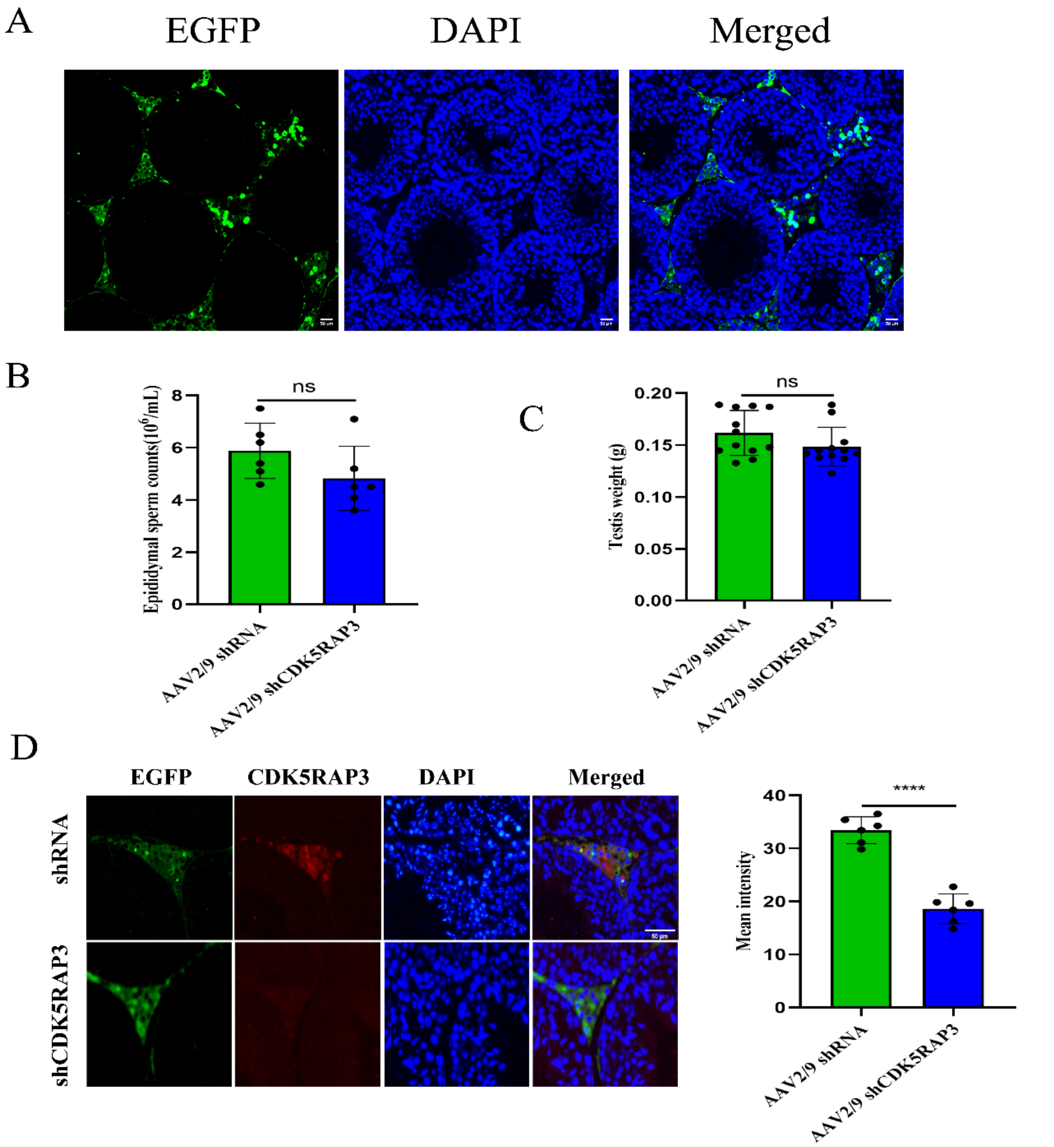

Figure S2. *In Vivo* injection results of AAV2/9-shCDK5RAP3. (n=6 per group) (A) Expression of EGFP in testicular Leydig cells. (B) Sperm count in the epididymis of mice. (C) Testis weight of mice. (D) Immunofluorescence results of CDK5RAP3 in mouse testes. scale bar, 50 µm. **** *P*<0.0001. Abbreviations: EGFP, enhanced green fluorescent protein; CDK5RAP3, cyclin-dependent kinase 5 regulatory subunit–associated protein 3.

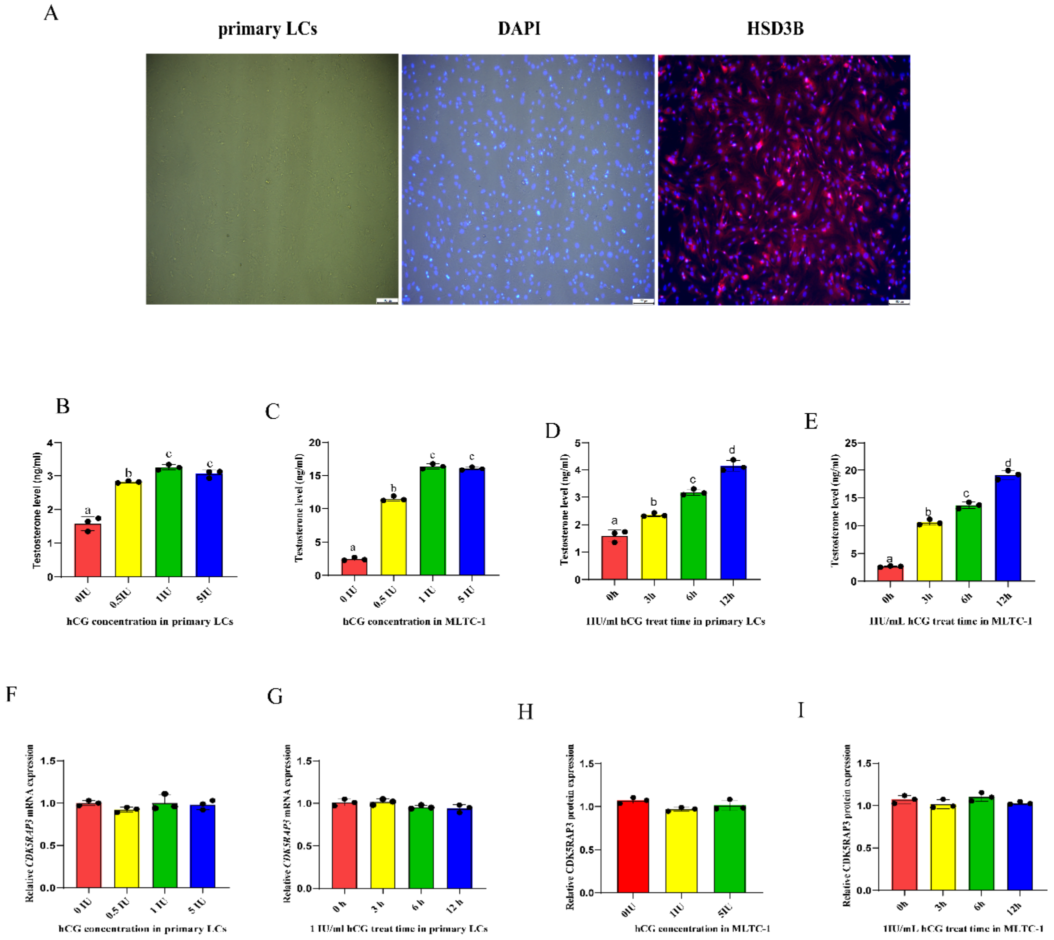

Figure S3. The effect of HCG on CDK5RAP3 expression in primary LCs and MLTC-1 cells. (A) Identification of HSD3B immunofluorescence Staining in Primary LCs. (B-C) The results of HCG on Testosterone Secretion in Primary LCs. (D-E) The results of HCG on Testosterone Secretion in MLTC-1 cells. (F-G) The mRNA results of CDK5RAP3 in Primary LC after treatment with HCG. (H-I) The mRNA results of CDK5RAP3 in MLTC-1 cells after treatment with HCG. Different letters indicate significant differences, *P*<0.05. Data represent at least three independent biological replicates. Abbreviations: hCG, human chorionic gonadotropin.

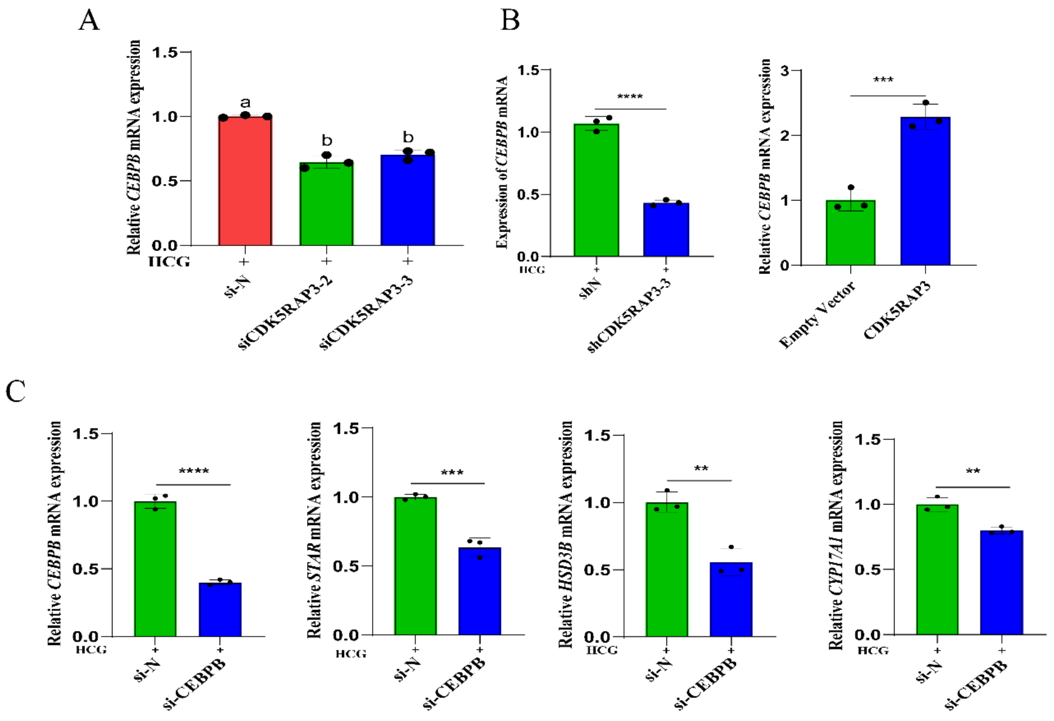

Figure S4. CDK5RAP3 regulate steroidogenic gene expression via interaction with CEBPB. (A) CEBPB mRNA levels in Primary LCs after si-CDK5RAP3 knockdown; (B) CEBPB mRNA levels in MLTC-1 cells after si-CDK5RAP3 knockdown and CDK5RAP3 overexpression. (C) The mRNA results of steroidogenic enzyme in MLTC-1 cells after si-CEBPB knockdown. * P <0.05. ** P <0.01. *** P <0.001. **** P <0.0001. Data represent at least three independent biological replicates. Abbreviations: CEBPB, CCAAT/enhancer-binding protein beta.

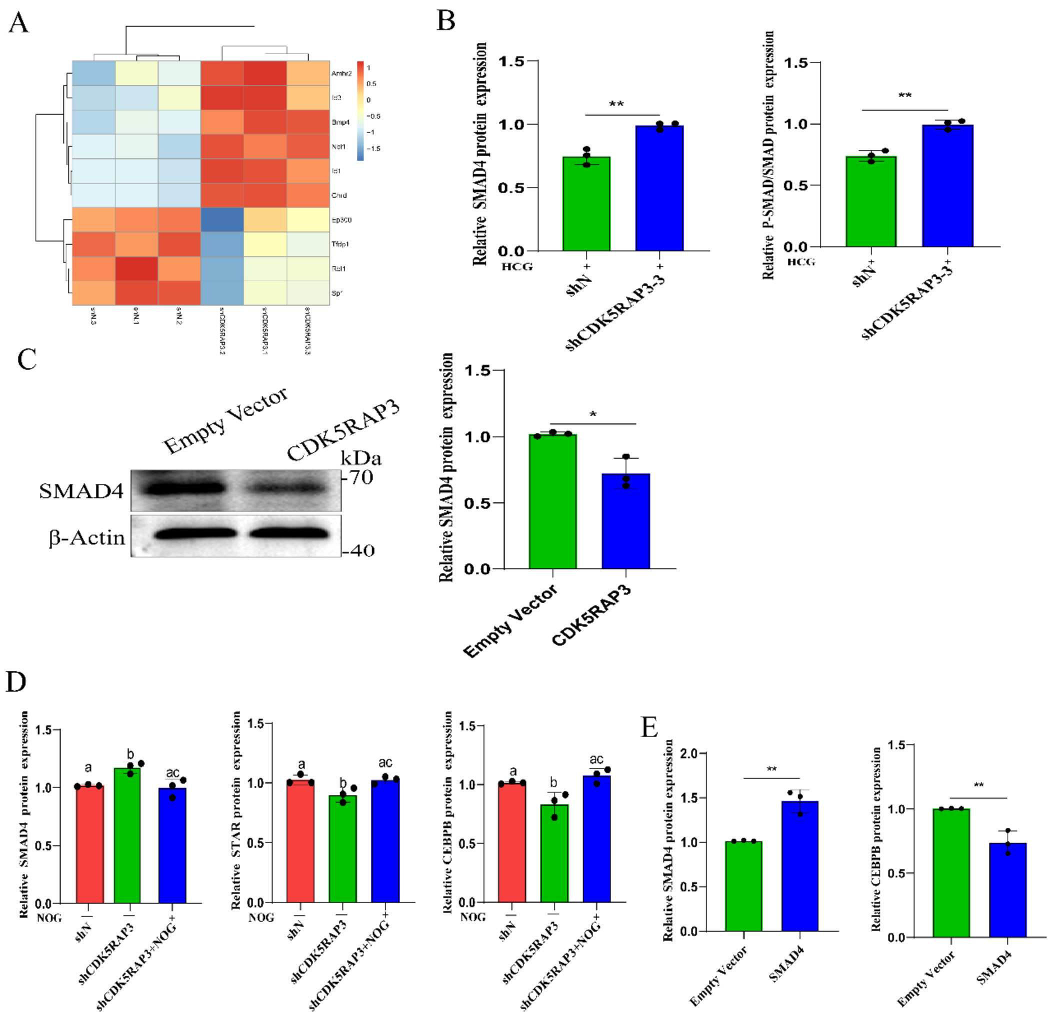

Figure S5. CDK5RAP3 regulates testosterone secretion through SMAD4/CEBPB axis. (A) Heat map of differential genes in TGF-beta signaling pathway. (B) Quantitative analysis of the western blot results of SMAD4 and P-SMAD1/5/9. (C) The western blot results of SMAD4 in CDK5RAP3 overexpression MLTC-1 cells. (D) Quantitative analysis of the western blot results in shCDK5RAP3 MLTC-1 cells treated with NOG. (E) Quantitative analysis of the western blot results in SMAD4 overexpression MLTC-1 cells. Different letters indicate significant differences, *P*<0.05. * *P* <0.05. ** *P* <0.01. *** *P* <0.001. **** *P* <0.0001. Data represent at least three independent biological replicates. Abbreviations: NOG, Noggin.

Table S1: qPCR primers used in this study

Gene symbol	Forward primer (5′→3′)	Reverse primer (5′→3′)	Amplicon size (bp)	Accession No.
Cdk5rap3	TCTGGCATCGTTGCTGAGACTC	TTCCTGTCTCCAGCACGGTGAT	139	NM_030248
Star	GTGCTTCATCCACTGGCTGGAA	GTCTGCGATAGGACCTGGTTGA	113	NM_011485
Cyp17a1	AGCTCTGTGCTGAACTGGATCC	AGACGGTGTTCGACTGAAGCCT	108	NM_007809
Cyp11a1	TGCTCAACCTGCCTCCAGACTT	ACTGGCTGAAGTCTCGCTTCTG	150	NM_019779
Hsd3b	ATCAGGGTCCTGGACAAGGTCT	TGGCAAGCTCTCCTCAGGTACT	128	NM_008293
Cebpb	CAACCTGGAGACGCAGCACAAG	GCTTGAACAAGTTCCGCAGGGT	113	NM_009883
β-Actin	CATTGCTGACAGGATGCAGAAGG	TGCTGGAAGGTGGACAGTGAGG	138	NM_007393
